# Editorial: Non-insulin pharmacotherapies for the treatment of type 2 diabetes and obesity - old and new players

**DOI:** 10.3389/fcdhc.2025.1747914

**Published:** 2025-12-08

**Authors:** Dimitrios Patoulias, Emir Muzurović, Manfredi Rizzo

**Affiliations:** 1 Second Propaedeutic Department of Internal Medicine, Faculty of Medicine, School of Health Sciences, Aristotle University of Thessaloniki, Thessaloniki, Greece; 2 Department of Internal Medicine, Endocrinology Section, Clinical Centre of Montenegro, Podgorica, Montenegro; 3 Faculty of Medicine, University of Montenegro, Podgorica, Montenegro; 4 College of Medicine, Ras Al Khaimah Medical and Health Sciences University, Ras Al Khaimah, United Arab Emirates; 5 School of Medicine, Promise Department, University of Palermo, Palermo, Italy

**Keywords:** type 2 diabetes, obesity, cardiovascular disease, SGLT-2 inhibitor, GLP-1RA

Type 2 diabetes (T2D) and obesity are major components of the so-called cardio-kidney-liver-metabolic syndrome, and they are closely interconnected, sharing common pathophysiologic mechanisms (1). They aggravate the overall prognosis of affected individuals, by increasing the risk of future cardio-renal complications (1). The increasing trends in the prevalence of both diseases are truly alarming, and the projections for the coming decades are pessimistic, predicting a substantial increase in the total number of affected individuals worldwide. Therefore, a tailored therapeutic approach is required in order to prevent or delay the progression of the disease and related complications that increase overall morbidity and mortality. Insulin remains the mainstay of treatment for difficult-to-treat and insulin-depleted T2D patients, especially in long-standing disease; however, current therapeutic approach focuses on early initiation of drug classes with established cardio-renal benefits, such as sodium-glucose co-transporter-2 inhibitors (SGLT-2i) and glucagon-like peptide-1 (GLP-1) receptor agonists (RAs) (2, 3), whereas the latest class has now been placed among the most efficacious pharmacological treatment option for individuals with obesity, even without concomitant T2D (4, 5).

This Research Topic investigated the role of both newer and established agents in the treatment of both conditions, highlighting their potential for affected individuals, with emphasis not only on gluco-metabolic efficacy but also on safety.

One of the main aspects that can affect treatment efficacy in T2D, which is often neglected but can result in treatment discontinuation, is the occurrence of drug-to-drug interactions, sometimes resulting in adverse drug reactions. In a relevant retrospective study from China, Ren et al. demonstrated that among hospitalized individuals with T2D, the prevalence of drug-to-drug interactions was 99.73% during hospitalization and 85.21% after discharge, with polypharmacy being the major determinant during hospitalization, whereas polypharmacy and the presence of diabetic complications represented the major contributing factors to drug-to-drug interactions after discharge. These findings highlight the need for an individualized therapeutic approach in T2D, with early recognition of patients at risk for adverse drug interactions and the rational use of different drug classes.

Another main determinant in the management of T2D, obesity, or both is the pattern of each individual’s eating behavior. GLP-1 RAs have revolutionized the treatment of T2D and obesity due to their multiple beneficial and pleiotropic effects, including anti-atherogenic benefit (6). Based on the results of a multicenter, prospective observational study conducted in Japan, Koide et al. found that 12-month treatment with GLP-1 RAs in 92 individuals with T2D and overweight/obesity led to significant improvement in glycemic control, along with significant reduction in body weight, body mass index and body fat percentage, while no change in skeletal muscle mass was observed. Of note, the researchers also demonstrated that treatment with GLP-1 RAs provoked a significant and sustained reduction in external eating scores both at 3- and 12-months post-treatment initiation, a factor linked to overeating and consequently to body weight gain among individuals with T2D. Most importantly, individuals with greater baseline external eating scores were shown to achieve greater body weight reduction at 12 months with GLP-1 RA treatment, highlighting external eating as an underexplored but significant predictor of the weight-lowering efficacy of this drug class.

Besides newer drug classes, which have completely revolutionized the present therapeutic strategy in T2D, insulin remains the ultimate pharmacological option for difficult-to-treat T2D, especially in cases of long-standing disease and severe insulin deficiency due to the progressive loss of function of pancreatic beta-cells. Insulin glargine U300, a basal insulin analogue administered on a daily basis, offers a sustained and stable insulin release thereby preventing substantial glycemic variability and providing adequate glycemic control. In a phase I, single-center, double-blind, cross-over, randomized trial, Li et al. revealed that a newer Gan & Lee (GL) glargine U300 shares a similar pharmacokinetic and pharmacodynamic profile with currently commercially available insulin glargine U300, as demonstrated through the utilization of the glucose clamp technique. The safety profiles of both products were also similar, although no immunogenicity assessment of the investigational product was performed. Overall, newer insulin analogues can enrich the therapeutic arsenal against T2D, in an era marked by the development of weekly basal insulin analogues that may offer significant flexibility for patients and enhance treatment adherence.

Except for pharmacological therapeutic options, non-pharmacological options for the treatment of T2D were also explored in this Research Topic. Odeniran et al. performed a thorough systematic review assessing the therapeutic efficacy of a plant widely distributed in Africa, named *Balanites aegyptiaca* DEL, in T2D. All eligible animal studies demonstrated that *Balanites aegyptiaca* DEL offers antidiabetic effects, leading to significant reduction in blood glucose levels whereas some studies also revealed that this agent may improve insulin sensitivity, lipid profile, and hepatic and renal function and may also promote body weight loss. Among other mechanisms of action, *Balanites aegyptiaca* DEL appears to exert significant anti-inflammatory and antioxidant effects, while also regulating glucose/carbohydrate metabolism and mitigating insulin resistance. However, human studies in the field are required to confirm this anti-diabetic efficacy of *Balanites aegyptiaca* DEL.

Overall, this Research Topic provides new and novel insights into the treatment of T2D and obesity, highlighting the absolute need for an individualized therapeutic approach, tailored according to an individual’s co-morbidities, concomitant medications, lifestyle, financial status, current glycemic control and treatment goals, according to the principles of evidence-based medicine.
